# Comparison of cadaveric and isomorphic virtual haptic simulation in temporal bone training

**DOI:** 10.1186/s40463-014-0031-9

**Published:** 2014-10-13

**Authors:** Dana Wong, Bertram Unger, Jay Kraut, Justyn Pisa, Charlotte Rhodes, Jordan B Hochman

**Affiliations:** Department of Otolaryngology Head and Neck Surgery, University of Manitoba, Winnipeg, Manitoba Canada; Clinical Learning and Simulation Facility, Department of Medical Education, Faculty of Medicine, University of Manitoba, Winnipeg, Manitoba Canada; Department of Medical Education, Faculty of Medicine, University of Manitoba, Winnipeg, Manitoba Canada; Surgical Hearing Implant Program, Department of Otolaryngology - Head and Neck Surgery, University of Manitoba, GB421, 820 Sherbrook Street, Winnipeg, Manitoba R3A 1R9 Canada; Neurotologic Surgery, Department of Otolaryngology Head and Neck Surgery, Faculty of Medicine, University of Manitoba, GB421, 820 Sherbrook Street, Winnipeg, Manitoba R3A 1R9 Canada

**Keywords:** Medical simulation, Haptic, Real time marching cubes, Temporal bone

## Abstract

**Background:**

Virtual surgery may improve learning and provides an opportunity for pre-operative surgical rehearsal. We describe a novel haptic temporal bone simulator specifically developed for multicore processing and improved visual realism. A position locking algorithm for enhanced drill-bone interaction and haptic fidelity is further employed. The simulation construct is evaluated against cadaveric education.

**Methods:**

A voxel-based simulator was designed for multicore architecture employing Marching Cubes and Laplacian smoothing to perform real-time haptic and graphic rendering of virtual bone.

Ten Otolaryngology trainees dissected a cadaveric temporal bone (CTB) followed by a virtual isomorphic haptic model (VM) based on derivative microCT data. Participants rated 1) physical characteristics, 2) specific anatomic constructs, 3) usefulness in skill development and 4) perceived educational value. The survey instrument employed a Likert scale (1-7).

**Results:**

Residents were equivocal about the physical properties of the VM, as cortical (3.2 ± 2.0) and trabecular (2.8 ± 1.6) bone drilling character was appraised as dissimilar to CTB. Overall similarity to cadaveric training was moderate (3.5 ± 1.8). Residents generally felt the VM was beneficial in skill development, rating it highest for translabyrinthine skull-base approaches (5.2 ± 1.3). The VM was considered an effective (5.4 ± 1.5) and accurate (5.7 ± 1.4) training tool which should be integrated into resident education (5.5 ± 1.4). The VM was thought to improve performance (5.3 ± 1.8) and confidence (5.3 ± 1.9) and was highly rated for anatomic learning (6.1 ± 1.9).

**Conclusion:**

Study participants found the VM to be a beneficial and effective platform for learning temporal bone anatomy and surgical techniques. They identify some concern with limited physical realism likely owing to the haptic device interface. This study is the first to compare isomorphic simulation in education. This significantly removes possible confounding features as the haptic simulation was based on derivative imaging.

## Background

Current temporal bone surgical training is centered on graduated operative practice under the supervision of an experienced surgeon. As a corollary to increasing focus on safety, and to supplement surgical education in the face of resident work hour restrictions, numerous teaching adjuncts have been developed. The Cadaveric Temporal Bone Lab remains the gold standard; however access to sufficient exposure is site specific owing to local factors and expense [1]. An array of haptic simulators [2-10] are now available to complement this training and the field of additive manufacturing is beginning to provide effective models for dissection [11,12].

### Haptic simulation for surgical training

Haptic simulation provides real-time 3 dimensional contact force representation. The user sees a graphical representation of the bone and *feels* it using a manipulandum held in the hand in an analogous fashion to an otic drill. Movement of the manipulandum guides the virtual drill tip. As the virtual bone is drilled, deep structures are revealed, permitting simulated complex surgical procedures. While this does provide a sense of drill-bone interaction, the experience is not identical to that of operative drilling.

The advantages of haptic simulation are easy operation, the absence of biologic materials, the ability to provide a wide range of anatomic variants, failure without consequence, and provision for repeated practice. Perhaps the most significant advantage is the ability to objectively monitor and assess trainee actions, providing a basis for formative and summative metrics [13-18]. Further, there may be utility in competency based residency training.

These benefits have led to the development of numerous haptic surgical trainers [2-10]. The validity of haptic trainers has been studied, particularly with reference to surgical performance and construct validity [13,15-17]. Direct comparison to performance in standard cadaveric dissection [12,13] have previously shown mixed results. These studies appear to have used a standardized haptic model which was compared to anatomically unmatched cadaveric samples. In the study described below, unique isomorphic models of cadaveric bones were created so that participants drill anatomically identical bones in both modalities, eliminating anatomical variation as a confounding factor in analysis.

The haptic simulation of temporal bone which we use takes advantage of incremental gains in processing speed and computer architecture to generate contact forces using a novel algorithm [19].

### Haptic simulation of the temporal bone

**Figure 1:**
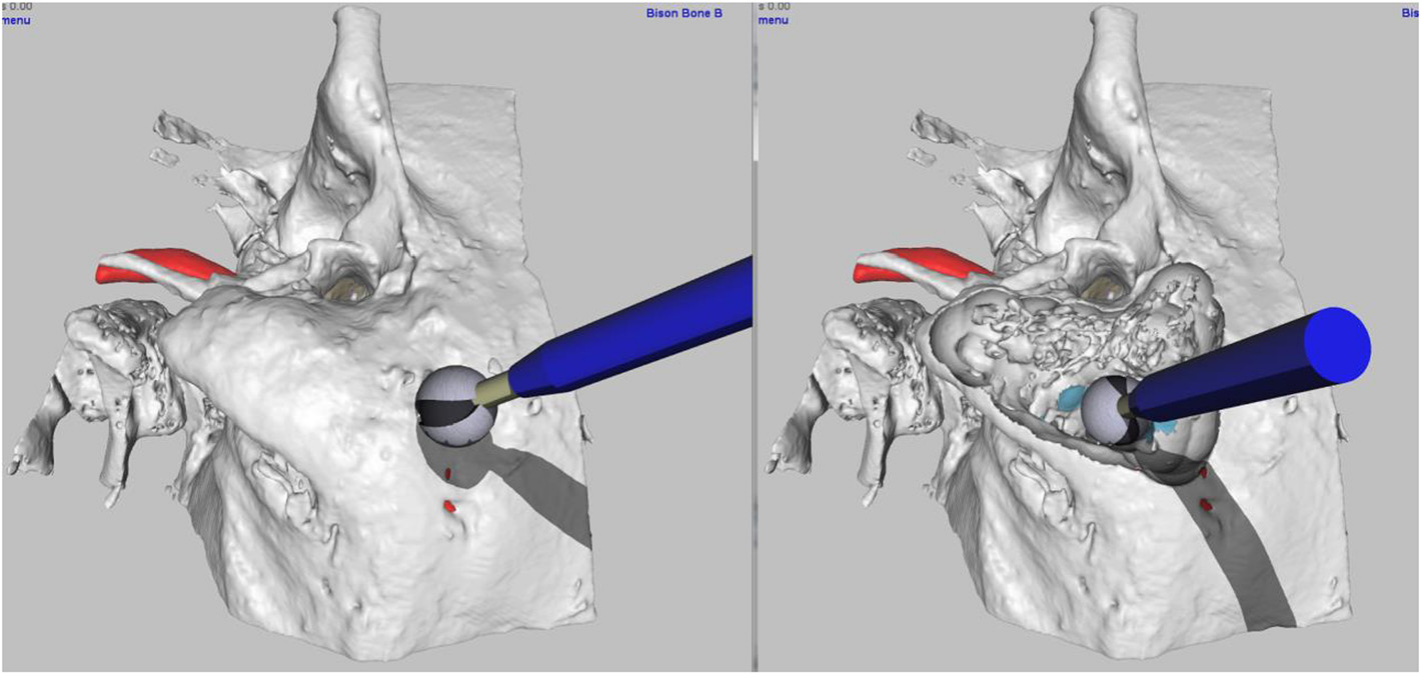
**Virtual temporal bone bimodal haptic graphic dissection.** Note the model does not appear voxellated and has excellent contours. The tympanic membrane (brown) sigmoid sinus (blue) and carotid artery (red) are apparent. The drill bit size is modifiable. The shadowing of the drill further facilitates appreciation of depth. The simulation is in 3D, employing active shutter glasses.

Temporal bone haptic simulation is not new. The earliest simulators converted voxel data to low resolution polygon surfaces [20] for display using volume rendering [21-23]. Our current simulation also uses voxel data for collision detection and force calculations, but renders the voxels graphically using high resolution polygons generated by the Marching Cubes algorithm [24] and Laplacian HC Smoothing [25]. These two algorithms run in real time using a new multicore architecture, creating a bone surface which appears smooth and free of step-like voxellation artifacts (Figure 1). The simulation runs on the Windows™ platform using DirectX™ which allows stereoscopic 3D using inexpensive consumer level 3D graphics cards and active shutter displays.

Our haptic display simulates forces felt by a simulated surgical drill. For the purposes of this study we used the inexpensive Phantom Omni device (Geomagic, Wilmington MA). The program is also compatible with 6 degree-of-freedom devices.

A position locking algorithm is used to calculate interaction forces rather than the more commonly used virtual spring methods [21]. This permits calculation of the location of the drill bit at every iteration and allows the haptic device to navigate fine surface features and improve stability when the drill tip is located in tightly constrained spaces.

The temporal bone haptic simulation we have developed employs CT data. The data is segmented into component structures, stored initially as individual polygon meshes which are then combined into a voxellated model for haptic display.

### Education centric platform

**Figure 2:**
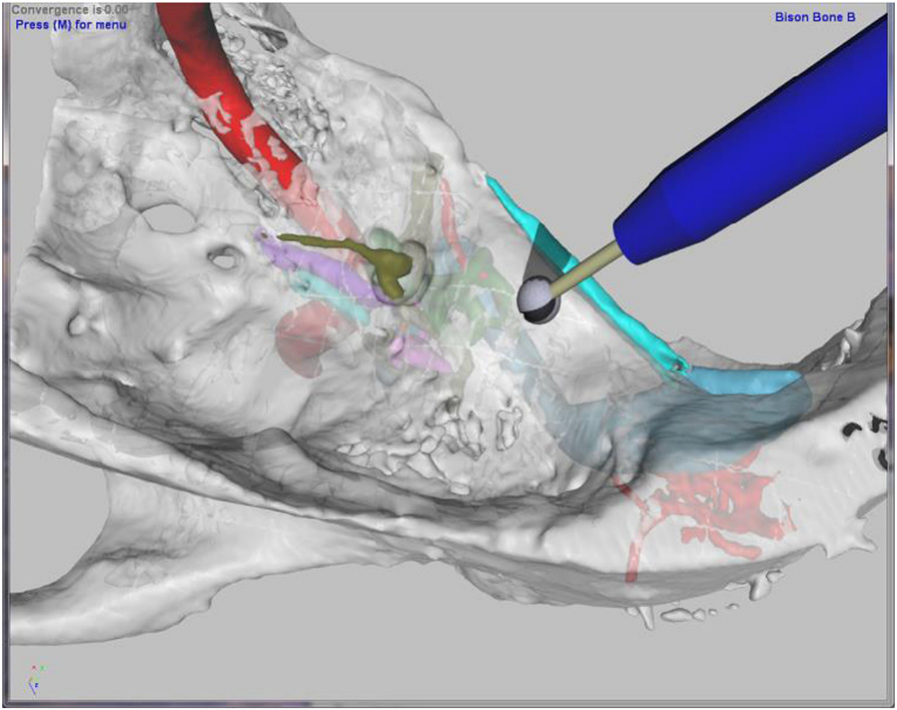
**Variable transparency in a Middle Fossa approach to the skull base.** The VM permits user exploration of approaches. Variable transparency allows for learning anatomy in disparate positions. Note the fidelity of the anatomy [Greater Superficial Petrosal Nerve and Geniculate Ganglion (olive), Superior Petrosal Sinus (turquoise) and Carotid Artery (red)]. The transparent function allows further appreciation of the anatomical relationships of structures [Sigmoid Sinus (blue), Emissary Vessels (red), and Superior Semicircular Canal (Green)].

**Figure 3:**
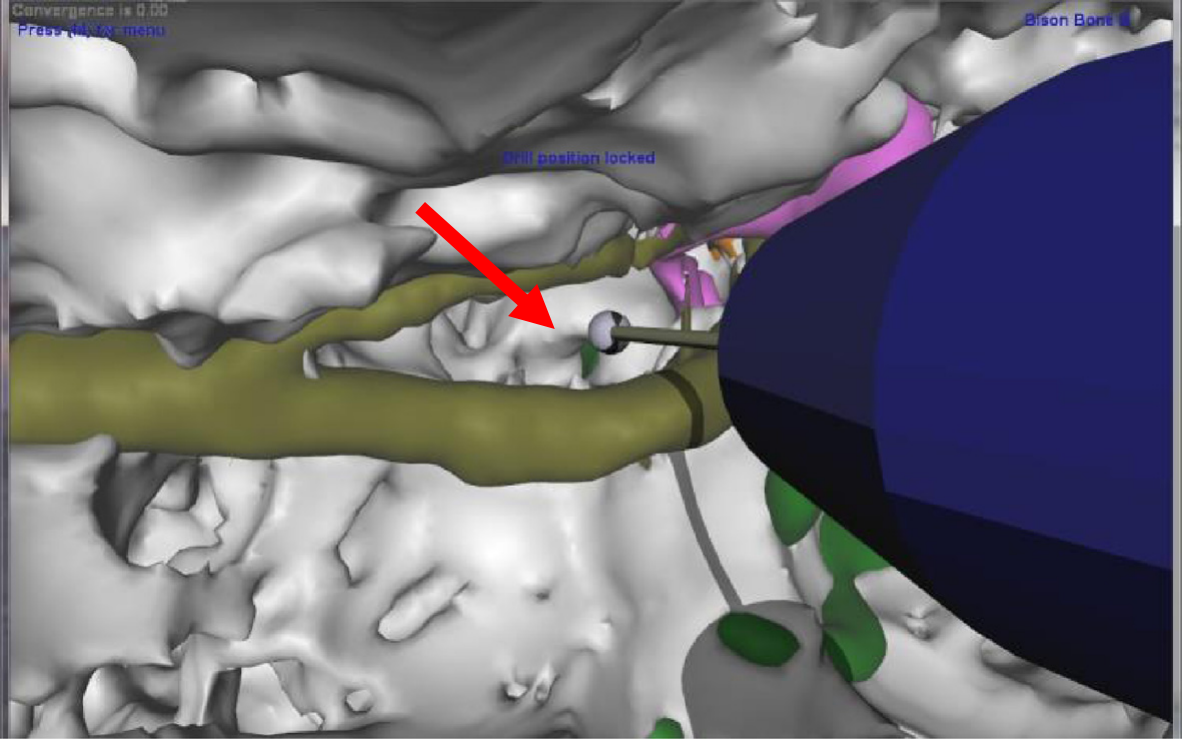
**Magnified posterior tympanotomy with visualization of the Round Window Membrane (RWM).** The bit size is reduced. Note the RWM (red arrow), vertical facial nerve and Chordae Tympani (olive) as well as ossicles (pink).

The purpose of the haptic simulation is to aid education. Software features included in the simulation permit drilling actions to be undone at the discretion of the user. Internal constructs can be made “undrillable” to facilitate learning the relative nature of anatomy. The ease of bone removal can be modified to aid in learning structure location. Two distinct training modes permit a user to both visually and manually follow an expert’s dissection of a bone model. The first is Passive Hand Motion Training. In this mode the user holds the haptic manipulandum while the computer replays the exact drill movements of an expert. The second mode is Active Hand Motion Training where arrows located in the upper right of the screen direct hand motion to closely replicate the expert’s recorded drilling process. Variable coloration, transparency, and stiffness of individual tissue components permit users to visualize anatomic structures more easily (Figures 2 and 3).

In the following, we describe the design of derivative haptic simulations from cadaveric temporal bone microCT data followed by experimental design, surgical resident preferences and perceptions of the model when compared directly to matched isomorphic cadaveric specimens.

## Methods

After study approval by the local Research Ethics Board (REB), ten residents each performed a cadaveric immediately followed by a virtual dissection of an isomorphic haptic model.

### Preparation of isomorphic haptic models from cadaveric specimens

Ten human cadaveric temporal bone specimens were prepared for otic drilling by resident surgical trainees. Prior to drilling, each bone underwent microCT using a SkyScan 1176 microtomograph (Bruker-microCT, Belgium). Image resolution was initially 35 μm but was down-sampled by a factor of 4 in x and y.

MicroCT data was then segmented using Mimics 14.0.1.7 (Materalize, Belgium) into separate anatomic features. Bone was segmented semi-automatically using Hounsfield unit thresholds. This ensured that void spaces such as air cells, were retained in the final model. Soft tissue features including carotid artery, sigmoid sinus, superior petrosal sinus, dural plates, endolyphatic sac, endolymphatic duct, otic capsule contents, ossicles, greater superficial petrosal, chordae tympani, facial nerves, cochleariform process and semi-canal for tensor tympani, were manually segmented. Segmented features were stored as individual polygon meshes.

A haptic simulation of each cadaveric specimen was then generated by recombining its individual polygon mesh models into a single voxellated model [19]. Each cadaveric bone specimen, therefore, had a corresponding haptic simulation which contained anatomy identical in size and shape (isomorphic) to the parent bone. The simulation used a haptic device (Geomagic Touch - SC, USA) to control a virtual drill during interaction with the voxellated model (Figures 1, 2 and 3). The model was visually displayed on a 165 cm plasma screen (Panasonic TCP65VT30, Panasonic, Osaka, Japan) mounted above and behind the haptic device (1280×720 pixel resolution). The drill was activated using an on-off foot-pedal (Scythe – Tokyo, Japan).

### Resident evaluation of haptic temporal bone models

10 surgical resident trainees, with varying degrees of surgical experience, from the Otolaryngology program at the University of Manitoba, gave informed consent for participation in the study. Each student was randomly assigned a cadaveric bone and its matched isomorphic haptic model for dissection. Subjects first drilled their assigned cadaveric specimen under supervision of a Neurotologist using an otic drill (Stryker, Michigan, USA). Following completion of cadaveric drilling, each subject drilled the isomorphic haptic model matching the cadaveric bone on which they had just practiced. No time limit on the session was set although all subjects completed cadaveric and virtual drilling in less than 4 hours. Subjects then completed a survey instrument (Likert Scale) comparing haptic and cadaveric drill experiences. The survey asked subjects to rate the haptic model in four areas as compared to cadaveric dissection, including 1) physical characteristics of the VM, 2) specific anatomic feature representation of the VM 3) usefulness in surgical skills training and 4) perceived educational value. A copy of the survey instrument can be obtained from the corresponding author.

## Results

The mean and standard deviation of resident responses can be seen in the tables below for each of the four survey components.

Residents were ambivalent about the physical similarity of the VM as compared to CTB (Table 1), rating it highest for its air cell system representation (5.4 ± 1.4). Hardness was rated better for cortical (3.2 ± 2.0) than trabecular (2.8 ± 1.6) bone, but neither was considered similar to CTB. The simulations overall physical similarity to CTB (3.5 ± 1.8) was unexceptional.

**Table 1:** **Resident assessment of virtual model physical properties as compared to cadaveric bone**

**Model factor**	**Mean comparison rating ± SD (1 = very dissimilar, 7 = very similar)**
**Cortical bone hardness**	3.2 ± 2.0
**Trabecular bone hardness**	2.8 ± 1.6
**Vibrational properties**	3.2 ± 1.5
**Acoustic properties**	2.7 ± 2.0
**Drill skip**	2.9 ± 2.0
**Air cell system**	5.4 ± 1.4
**Thinning of dural plates**	3.5 ± 1.8
**Palpation of Dura**	2.2 ± 1.6
**Overall similarity to CTB**	3.5 ± 1.8

Residents generally rated the VM’s internal constructs as more similar to CTB than its physical properties (Table 2), with the highest values awarded to vascular structures (range 5.6 to 5.8) and the lowest to dural plates (4.5 ± 1.7). Important middle ear, otic capsule and nervous structures were reasonably considered (range 5-5.5).

**Table 2:** **Resident assessment of virtual model anatomical feature similarity to cadaveric bone**

**Anatomical feature**	**Mean similarity rating ± SD (1 = very dissimilar, 7 = very similar)**
**Mastoid/vertical facial nerve**	5.4 ± 1.4
**Tympanic/horizontal facial nerve**	5.5 ± 1.4
**Dural plates**	4.5 ± 1.7
**Dura**	4.5 ± 1.5
**Incus**	5.3 ± 1.4
**Stapes**	5.3 ± 1.3
**Malleus**	5.2 ± 1.3
**Horizontal SCC**	5.0 ± 1.4
**Posterior SCC**	5.0 ± 1.4
**Superior SCC**	5.0 ± 1.4
**Carotid Artery**	5.6 ± 0.9
**Sigmoid sinus**	5.8 ± 1.1
**Cochleariform process**	5.3 ± 0.7
**Facial recess**	5.2 ± 1.2
**Sinus Tympani**	5.2 ± 1.0
**Internal auditory canal**	5.3 ± 1.2

Residents generally felt that the VM was beneficial in surgical skill acquisition (Table 3), rating it highest for translabyrinthine approaches to the skull base (5.2 ± 1.3) and lowest for sigmoid sinus decompression (4.4 ± 2.0). All surgical skills assessed were deemed to benefit from training on the VM.

**Table 3:** **Resident perceived value of virtual model in surgical skill acquisition**

**Surgical skill**	**Mean value ± SD (1 = not beneficial, 7 = very beneficial)**
**Canal wall up mastoidectomy**	4.9 ± 1.7
**Facial recess approach**	4.8 ± 1.8
**Canal wall down mastoidectomy**	5.1 ± 1.4
**Inside out mastoidectomy**	4.7 ± 1.8
**Bondy mastoidectomy**	4.8 ± 1.6
**Sigmoid sinus decompression**	4.4 ± 2.0
**Dural plate decompression**	4.6 ± 2.0
**Labyrinthectomy**	5.0 ± 1.5
**Translabyrinthine approach to IAC**	5.2 ± 1.3
**Middle fossa approach to IAC**	4.7 ± 1.8
**Middle fossa approach to superior canal dehiscence**	5.0 ± 1.5

Residents generally agreed that the VM was an effective (5.4 ± 1.5) and accurate (5.7 ± 1.4) tool which should be integrated into education (5.5 ± 1.4) (Table 4). Participants did not consider the VM a viable replacement of CTB dissection (2.5 ± 2.3). Generally, the VM was presumed to increase surgical performance (5.3 ± 1.8) and confidence (5.3 ± 1.9) and was ranked highly with respect to its usefulness in teaching anatomy (6.1 ± 1.9) and facilitating access to a broad range of pathologic and anatomic variation (5.6 ± 1.8).

**Table 4:** **Resident appraisal of virtual model educational value**

**Educational evaluation statement**	**Mean agreement level ± SD (1 = strongly disagree, 7 = strongly agree)**
**This is an effective training instrument**	5.4 ± 1.5
**This instrument is an accurate reproduction of the temporal bone**	5.7 ± 1.4
**This instrument should be integrated into resident education**	5.5 ± 1.4
**This form of simulation can replace the cadaveric temporal bone lab**	2.5 ± 2.3
**This simulation provides a basis for appreciating the relative anatomy of temporal bone structures**	6.1 ± 1.9
**This simulated surgery improves confidence**	5.3 ± 1.9
**Increased exposure to this simulation would improve resident surgical performance**	5.3 ± 1.8
**Increased exposure to this simulation would improve resident comfort with actual patient surgery**	5.4 ± 1.8
**This simulation facilitates practice of skills across a range of anatomical and pathologic variations (sclerotic, low dura, disease)**	5.6 ± 1.8

## Discussion

This is a first description of a novel multicore haptic temporal bone simulation employing a position locking algorithm and validated using isomorphic models. The simulation allows for multiple segmented models to be created in the formation of a virtual library.

Participants feel the haptic simulation is beneficial in learning surgical skills and neurotologic surgical approaches. Residents found the haptic simulation to be an effective teaching platform with favorable internal anatomic representation.

Problematic drill character is due to the inability of the haptic device to render a stiff bone surface with realistic drill vibration. While improving digital processing and graphic representation, a multicore design has limited impact on the effectiveness of the manipulandum. The change to a position locking algorithm, realizes improved processing; however, based on these results, does not further advance drill experience. A direct comparison to a virtual spring haptic system was not undertaken, but may prove useful to determine differences in the user experience.

This study has several advantages. The most noteworthy is the use of an isomorphic haptic model, derived directly from the template CTB. Previous studies have focused on comparing generic cadaveric dissection to haptic simulation [13,14]. Cadaveric dissection irreversibly destroys the anatomy of the specimen. By preserving the specimen’s anatomy in the form of a haptic model, it is possible to make direct comparison of the simulation’s effectiveness without the confounding issue of differing anatomical features between the studied modalities. The preservation of the anatomy also permits repeated testing on the same specimen and the potential for developing large digital libraries.

The most serious study limitation is its small sample size and single centre nature. While the study examined all ENT surgery residents at the University of Manitoba, it is possible that institutional bias may have influenced the findings. A more rigorous multi-centre trial design with a carefully defined curricular program, looking at resident and expert perceptions, as well as performance metrics and clinical outcomes is currently being designed.

Improved training may reduce patient risk exposure. Simulated temporal bone training can address needs in continuing education, competency based residency training, and ultimately become a component of the certification process. We currently have a large and expanding library based on microCT data which we hope to utilize for these purposes.

## Conclusion

We describe a novel haptic temporal bone simulator (VM) derived from imaging of cadaveric bone. We evaluated our VM against the derived cadaveric bone. Study participants found that the VM was both a beneficial and an effective platform for learning temporal bone anatomy and surgical techniques. They also identify some concern with limited physical realism likely owing to the haptic device interface. Virtual surgery may improve learning and provide added opportunity for pre-operative surgical rehearsal without comparison patient safety. This study is the first to compare isomorphic simulation in education.

## Abbreviations

CTB: Cadaveric temporal bone
VM: Virtual isomorphic haptic model
